# The Ubiquitin E3 Ligase Nedd4 Regulates the Expression and Amyloid-β Peptide Export Activity of P-Glycoprotein

**DOI:** 10.3390/ijms23031019

**Published:** 2022-01-18

**Authors:** Amanda B. Chai, Richard Callaghan, Ingrid C. Gelissen

**Affiliations:** 1 School of Pharmacy, Faculty of Medicine and Health, University of Sydney, Sydney, NSW 2006, Australia; acha3237@uni.sydney.edu.au; 2 School of Biomedical Sciences, Faculty of Biological Sciences, University of Leeds, Leeds LS2 9JT, UK; r.callaghan@leeds.ac.uk

**Keywords:** Nedd4, ubiquitin E3 ligase, P-glycoprotein, ABCB1, amyloid-beta

## Abstract

The ATP-binding cassette transporter, P-glycoprotein (P-gp), has been demonstrated to facilitate the clearance of amyloid-beta (Aβ) peptides, exporting the neurotoxic entity out of neurons and out of the brain via the blood–brain barrier. However, its expression and function diminish with age and in Alzheimer’s disease. P-gp is known to undergo ubiquitination, a post-translational modification that results in internalisation and/or degradation of the protein. NEDD4-1 is a ubiquitin E3 ligase that has previously been shown to ubiquitinate P-gp and reduce its cell surface expression. However, whether this effect translates into altered P-gp activity remains to be determined. siRNA was used to knockdown the expression of Nedd4 in CHO-APP cells. Western blot analysis confirmed that absence of Nedd4 was associated with increased P-gp protein expression. This was accompanied by increased transport activity, as shown by export of the P-gp substrate calcein-AM, as well as enhanced secretion of Aβ peptides, as shown by ELISA. These results implicate Nedd4 in the regulation of P-gp, and highlight a potential approach for restoring or augmenting P-gp expression and function to facilitate Aβ clearance from the brain.

## 1. Introduction

Amyloid-β (Aβ) peptides are a normal cellular product generated by the routine enzymatic cleavage of amyloid precursor protein (APP). In healthy individuals, these peptides are efficiently cleared from the brain via cellular uptake by astrocytes, neurons and glial cells, enzymatic degradation, and export into peripheral circulation [1,2]. However, impairments to these clearance mechanisms are associated with the pathological deposition and accumulation of Aβ peptides observed in the brains of Alzheimer’s patients [3]. Aβ peptide monomers readily aggregate to form oligomers and fibrils, with the former considered to be the most significant factor leading to neurotoxicity, synaptic dysfunction and memory impairment, and the latter constituting the hallmark amyloid plaques in Alzheimer’s disease (AD) [4,5]. It is estimated that 25–60% of Aβ peptides are cleared from the brain via active transport across the blood–brain barrier (BBB) [6,7,8], and multiple lines of evidence have highlighted the involvement of the 170 kDa ATP-binding cassette (ABC) transport protein ABCB1, also known as P-glycoprotein (P-gp), in this process [9]. P-gp has been demonstrated to interact directly with Aβ peptides [10,11,12] and mediate their export from BBB endothelial cells as well as neuronal cells, where the majority of these peptides are synthesised [10,13,14]. Studies in humans have reported inverse correlations between P-gp expression and function with age [15,16], Aβ deposition [15,17,18], and AD [19,20,21]. However, the temporality and precise mechanisms underlying these relationships remain unclear. 

Post-translational modification presents a salient aspect of protein regulation. By engendering complexity and diversification of protein expression and activity beyond what is encoded by the genome, these modifications determine the ultimate biological function of the protein. Ubiquitination is one such post-translational modification that P-gp has been demonstrated to undergo [22,23]. Intriguingly, it has been suggested that Aβ peptides may downregulate P-gp activity by promoting its ubiquitination and subsequent degradation, thereby exacerbating Aβ deposition within the brain [24]. Ubiquitination involves the tagging of a target protein with a small 76 amino acid polypeptide known as ubiquitin, via the sequential activities of three enzymes. The process begins when ubiquitin activating enzyme (E1) activates a ubiquitin monomer and transfers it to the ubiquitin conjugating enzyme (E2) via formation of thioester bonds. The E2 enzyme then transfers ubiquitin to ubiquitin ligase (E3), which covalently links the ubiquitin moiety to exposed lysine residues of the target protein [25]. Proteins may be mono- or poly-ubiquitinated, depending on the length and topology of the linked ubiquitin chain. The tagged protein may subsequently be recognized by the 26S proteasome for degradation, or targeted for lysosomal degradation, both mechanisms of which have been demonstrated for P-gp [22,26,27]. Furthermore, P-gp in the plasma membrane may also undergo endocytosis and recycling [28]. Specificity of the ubiquitination process is determined by the E3 ligase, of which 600–700 have been identified in humans thus far [29]. Although several E3 ligases have been identified to regulate P-gp expression in cancer cells [27,30,31], only one has been investigated with respect to P-gp located in the brain. Neural precursor cell-expressed developmentally downregulated protein 4-1 (NEDD4-1), a ubiquitously expressed protein and founding member of the NEDD4 family of E3 ligases, has been demonstrated to recognize and interact with P-gp [32]. NEDD4-1 contains an N-terminal C2 domain that enables phospholipid membrane binding, four tryptophan-containing WW domains for substrate recognition, and a C-terminal Homologous to the E6-AP Carboxyl Terminus (HECT) domain that catalyses the transfer of ubiquitin to the target protein [33]. Using recombinant proteins, Akkaya et al. showed that NEDD4-1 directly tags eight lysine residues on the cytosolic surface of P-gp with ubiquitin, resulting in reduced plasma membrane expression of the transporter [32].

In this investigation, we sought to expand upon the present understanding of the NEDD4-1/P-gp relationship, and hypothesise that downregulating NEDD4-1 could enhance P-gp expression and activity in the brain. Utilising an in vitro approach, we examined the effects of the knockdown of Nedd4 (rodent homologue of human NEDD4-1) on P-gp protein expression, transport function, and Aβ peptide transport capacity in Chinese hamster ovary cells stably overexpressing APP (CHO-APP). Our data suggest that Nedd4 is implicated in the post-translational regulation of P-gp, and that enhancing P-gp function by modulating Nedd4 could facilitate Aβ clearance from the brain.

## 2. Results

### 2.1. Knockdown of Nedd4 Enhances P-gp Protein Expression

**Figure 1:**
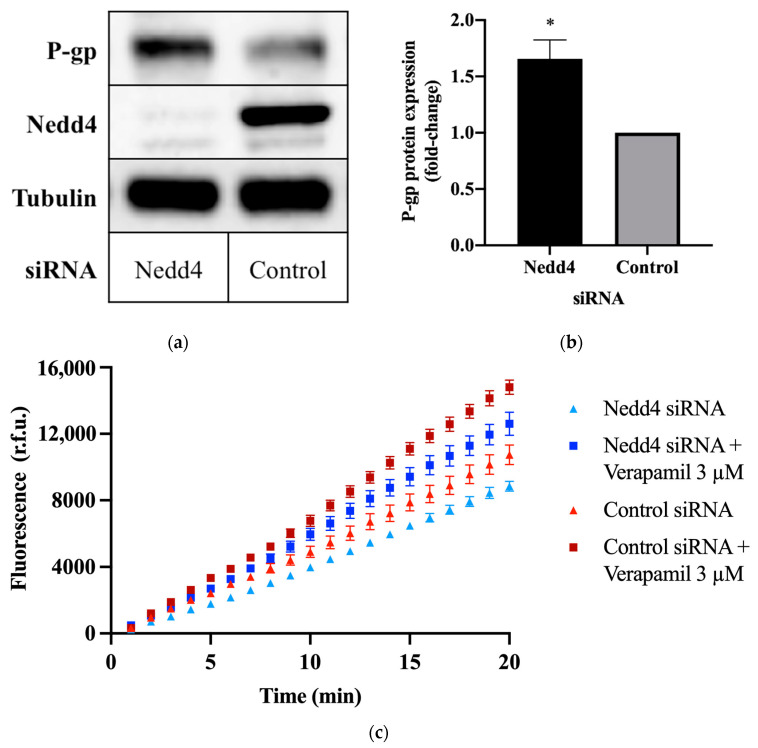
Effect of Nedd4 knockdown on P-gp protein expression and activity in CHO-APP cells. (**a**) CHO-APP cells were transfected with Nedd4 siRNA or universal control for 48 h. Immunoblots are representative of three independent experiments. The housekeeping protein, tubulin, was used as a loading control. (**b**) Relative expression of P-gp protein in lysates of CHO-APP cells treated with Nedd4 siRNA or universal control. P-gp signal intensities were normalised to tubulin. Data (mean ± SEM of three independent experiments) were statistically analysed using the two-tailed Student’s *t*-test. * *p* ≤ 0.05 compared with control. (**c**) Intracellular accumulation of fluorescent calcein in CHO-APP cells transfected with Nedd4 siRNA or universal control for 48 h. Verapamil (3 μM), a P-gp inhibitor, was used as a positive control and added after the 48 h siRNA transfection and immediately before the calcein-AM assay was conducted. Data (mean ± SEM) are representative of three independent experiments, each performed with eight biological replicates.

To investigate the potential effect of Nedd4 on P-gp protein expression, CHO-APP cells were transfected with small interfering RNA (siRNA) against Nedd4. Western blots confirm that the knockdown of Nedd4 expression was indeed associated with a 1.6 ± 0.15-fold increase in P-gp protein expression, as compared with universal control siRNA-transfected cells (Figure 1a,b).

### 2.2. Knockdown of Nedd4 Increases P-gp Transport Activity

The calcein-acetoxymethyl ester (calcein-AM) assay was used to assess P-gp transport activity. Calcein-AM is a non-fluorescent membrane permeant dye and substrate of P-gp. In the presence of active P-gp, calcein-AM is efficiently removed from the cell before cleavage by intracellular esterases. If P-gp is inactive, calcein-AM is hydrolysed to calcein, which cannot be transported by P-gp and remains trapped within the cell, where it produces an intense fluorescence. Thus, the detected fluorescence intensity is inversely proportional to P-gp activity. This is demonstrated by the increased signal intensity (Figure 1c), and increased rate of intracellular calcein accumulation (Table 1) observed upon treatment with the P-gp inhibitor, verapamil. Conversely, knockdown of Nedd4 expression in CHO-APP cells was accompanied by a decrease in fluorescence (Figure 1c), and an increase in the rate of P-gp-mediated export of calcein-AM (Table 1) compared with control.

### 2.3. Knockdown of Nedd4 Promotes P-gp-Mediated Export of Aβ Peptides

**Figure 2:**
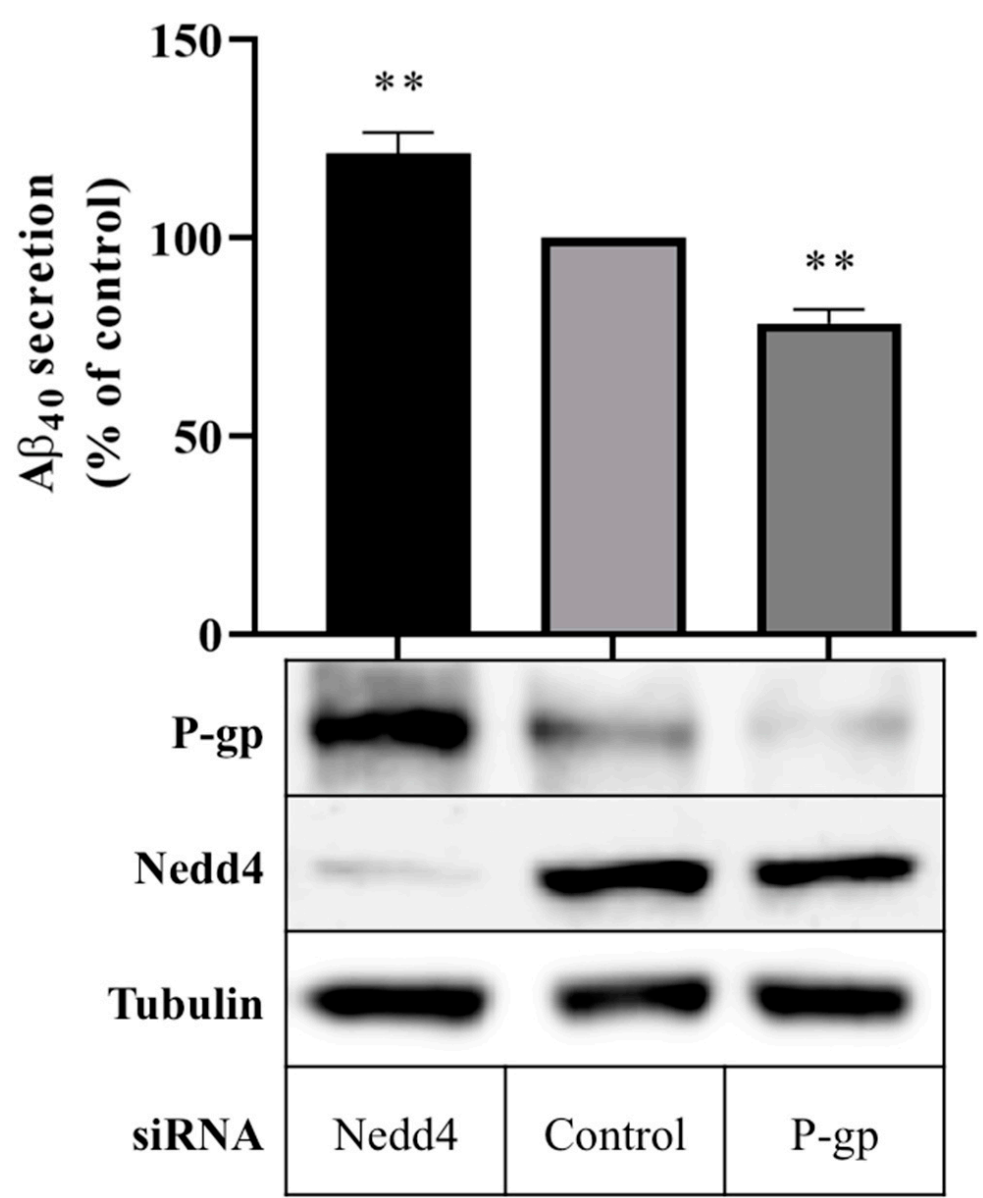
Effect of Nedd4 knockdown on Aβ export from CHO-APP cells. CHO-APP cells were transfected with Nedd4 or P-gp siRNA or universal control. Following the initial 24 h transfection period, transfection media was changed to fresh full culture media. After a further 24 h incubation period, cell media were collected for quantification of Aβ_40_ content by ELISA, and cells were harvested for Western blot analysis. The column graph shows the relative secretion of Aβ_40_ peptides into media over 24 h compared with control. Immunoblots confirm the knockdown of Nedd4 and enhancement of P-gp protein expression in Nedd4-siRNA treated cells compared with control. Tubulin was used as a loading control. Data reflect the mean ± SEM of three independent experiments. ** *p* ≤ 0.01 compared with control.

Aβ_40_ peptide secretion in cells expressing Nedd4 was compared with cells in which Nedd4 expression was silenced using a quantitative enzyme-linked immunosorbent assay (ELISA). Figure 2 shows that treatment with Nedd4 siRNA yielded a 21.3 ± 5.3% increase in Aβ_40_ secretion into cell media compared with control. In cells treated with P-gp siRNA, a 21.6 ± 3.5% reduction in Aβ_40_ secretion was observed. Western blot data confirm that P-gp protein expression was enhanced in the absence of Nedd4, and downregulated in the presence of P-gp siRNA (Figure 2).

## 3. Discussion

The involvement of the ubiquitin E3 ligase, NEDD4-1, in the post-translational regulation of P-gp was first identified by Akkaya et al. [32]. The authors demonstrated that NEDD4-1 directly attaches ubiquitin to P-gp, and that expression of NEDD4-1 in Flp-In HEK293 cells reduced the surface expression of P-gp [32]. Data presented here support these findings, implicating Nedd4 not only in the regulation of P-gp expression, but also, importantly, in the regulation of P-gp activity. In vitro techniques were used to substantiate the inverse relationship, wherein reducing Nedd4 expression was shown to enhance the protein expression and transport function of P-gp. Moreover, reducing Nedd4 was demonstrated to promote the P-gp-mediated export of Aβ_40_ peptides, as evidenced by the extent of the increase in Aβ_40_ peptide secretion by CHO-APP cells in the absence of Nedd4 corresponding to the extent of the decrease in Aβ_40_ peptide secretion in the absence of P-gp (Figure 2). These novel data suggest that modulating Nedd4 can markedly influence Aβ_40_ peptide secretion.

Data presented in Figure 1a and Figure 2 demonstrate that near-complete knockdown of Nedd4 increased P-gp protein expression by ~60%, and Aβ_40_ export by ~20%. Due to the complexities of cellular systems, it would be unlikely for protein expression and activity to be stoichiometrically linked. However, one avenue that warrants further investigation is the fate of P-gp following ubiquitination by NEDD4-1. Although there is evidence that ubiquitinated P-gp undergoes lysosomal and proteasomal degradation, the possibility of endosomal recycling (where the protein is expressed but inactive due to internalisation from the cell membrane) cannot yet be ruled out [22,26,34]. To this end, elucidating whether P-gp undergoes mono- or poly-ubiquitination by NEDD4-1, and investigating the effects of NEDD4-1 knockdown on cell surface versus total cell P-gp expression using cell surface biotinylation techniques, could be employed.

NEDD4-1 is expressed throughout the body, but has been shown to be particularly important for neuronal development and function [35,36]. Considering the neuron is a key site of synthesis for Aβ peptides, and recent evidence indicating that P-gp is able to export Aβ from neuronal cells, ascertaining whether NEDD4-1 additionally regulates P-gp expression and function at this locale could yield pathophysiological significance.

Since the seminal discovery that Aβ is a substrate of P-gp by Lam et al. in 2001 [37], many subsequent in vivo, ex vivo, in vitro, and molecular models have corroborated the ability for P-gp to export Aβ (reviewed in [9]). In addition, impaired P-gp expression and function have been identified alongside increased Aβ deposition in human and murine brains, and in patients with AD [9]. Hence, the observation by Akkaya et al. that exposure of isolated murine brain capillaries to Aβ_40_ peptides caused a 42 ± 5.7% increase in expression of Nedd4, and a concurrent 42 ± 0.5% reduction in expression of Abcb1, presents a potential mechanism by which Aβ clearance could be impaired in AD, a cyclical process that is aggravated by Aβ itself [32]. It remains to be established whether NEDD4-1 expression and functionality are altered with age or disease, however it has been reported that levels of ubiquitinated P-gp are significantly greater in the brains of patients with AD than non-AD subjects [23]. Nevertheless, data presented here impart further dimensionality to the interplay between NEDD4-1, P-gp, and Aβ, propounding that pharmacological inhibition of NEDD4-1-mediated ubiquitination of P-gp could serve as a strategy for restoring impaired P-gp expression and Aβ clearance from the Alzheimer’s brain.

## 4. Materials and Methods

### 4.1. Materials

All cell culture reagents, including media and additives, were purchased from Thermo Fisher Scientific (Scoresby, VIC, Australia), with the exception of Hanks’ Balanced Salt Solution (HBSS), which was purchased from Sigma-Aldrich (Castle Hill, NSW, Australia).

IGEPAL, protease inhibitor cocktail, dimethyl sulfoxide (DMSO), phosphate buffered saline (PBS), calcein-AM and verapamil hydrochloride were purchased from Sigma-Aldrich. Calcein-AM and verapamil powder were diluted in DMSO to produce stock solutions of 4 μM and 25 μM, respectively, and stored at −20 °C.

Transfection reagents, including Opti-MEM reduced serum medium and Lipofectamine RNAiMAX, were from Thermo Fisher Scientific. siRNA primers, including Mission siRNA targeting *ABCB1* (SASI_Hs01_00087519), custom-designed siRNA targeting hamster *Nedd4* (target sequence CTATGAATGGATTTGCTGA; validated in [38]), and universal negative control siRNA, were from Sigma-Aldrich.

Reagents for casting SDS-PAGE gels, including Tris-HCl, sodium dodecyl sulfate, and tetramethylethylenediamine were obtained from VWR Life Science (Tingalpa, QLD, Australia). 40% acrylamide/bis-acrylamide solution was from BioRad (Gladesville, NSW, Australia). Ammonium persulfate was from Sigma-Aldrich. Nitrocellulose membranes and enhanced chemiluminescent reagents were from Merck Millipore.

Anti-P-gp monoclonal antibody was from Thermo Fisher Scientific (MA5-13854). Anti-NEDD4-1 polyclonal antibody was from BioRad (VPA00462). Anti-tubulin monoclonal (T5168), and secondary anti-mouse (A9044) and anti-rabbit (A0545) antibodies were from Sigma-Aldrich.

### 4.2. Cell Culture

CHO-APP cells stably expressing human 695-amino acid APP cDNA were generated and maintained as described in [10].

### 4.3. siRNA Transfection

CHO-APP cells were seeded one day in advance to achieve 30–40% confluency on the day of the transfection. siRNA-lipid complexes were prepared by combining Nedd4, P-gp, or universal control siRNA, and Lipofectamine RNAiMAX at a ratio of 1:3 in Opti-MEM reduced-serum medium, achieving a final siRNA concentration of 0.094 μM. Cells were washed once with PBS, and then the medium from each well was replaced with antibiotic-free culture medium plus 200 µL (for 12-well plate format) or 100 µL (for 24-well format) transfection mixture. Cells were incubated at 37 °C for 48 h before harvest.

### 4.4. Calcein-AM Transport Assay

CHO-APP cells were seeded onto a 12-well plate in full culture medium and allowed to adhere overnight. The next day, cells were transfected with siRNA, as described in Section 4.3. Following an initial 24 h incubation, cells were rinsed once with PBS, trypsinised, centrifuged at 1000× *g* for 5 min, re-suspended in full culture medium, re-seeded onto a black-walled clear-bottomed 96-well plate at 4 × 10^4^ cells/well, and incubated at 37 °C for a further 24 h. On the day of the experiment, cells were washed once with phenol red-free HBSS. With or without verapamil (final concentration 3 μM), 100 μL of HBSS was added to each well using a multi-channel pipette, followed by 100 μL of calcein-AM substrate (final concentration 0.1 μM) in HBSS. Fluorescence measurements were taken every minute for 20 min, commencing immediately after the addition of calcein-AM, using the Perkin Elmer Victor X plate reader. The excitation and emission wavelengths were set at 485 and 535 nm, respectively. The plate reader temperature was maintained at 37 °C. Measurements were recorded in relative fluorescence units (RFU) and analysed using Graphpad Prism software (v6.01, La Jolla, CA, USA).

### 4.5. Cell Harvest and Western Blot

Cells were washed twice with ice-cold PBS and lysed with ice-cold cell lysis buffer (50 mM Tris, 150 mM NaCl, 1% IGEPAL, pH 7.8) containing protease inhibitor cocktail (5 µL/mL). Following a freeze/thaw cycle, lysates were syringed with 23-gauge needles to shear cellular DNA and centrifuged at 12,000× *g* at 4 °C for 5 min to remove debris. The protein concentrations of the resultant supernatants were quantified using the Pierce bicinchoninic assay (Thermo Fisher Scientific) in accordance with manufacture instructions. Equal amounts of cell protein were loaded onto 10% (*v*/*v*) acrylamide gels for separation by SDS-PAGE. Proteins were transferred onto nitrocellulose membranes, blocked for 1 h using 5% (*w*/*v*) skim milk, then incubated with anti-ABCB1 (1:100), anti-Nedd4 (1:1000), or anti-tubulin (1:3,000) overnight at 4 °C. Membranes were rinsed with PBS-Tween (0.5% *v*/*v*), then incubated with HRP-conjugated secondary antibodies (1:10,000) for 1 h at room temperature, and then rinsed again with PBS-Tween. Protein bands were visualized via chemiluminescence and the Bio-Rad ChemiDoc imaging system, and quantified by densitometry using Image J (v2.0.0, NIH, Bethesda, MD, USA).

### 4.6. Aβ_40_ ELISA

CHO-APP cells were seeded onto 24-well plates and transfected with siRNA as described in Section 4.3. Following the initial 24 h of transfection, transfection media was replaced with 500 μL/well of full culture media to enable accumulation of Aβ peptides over the subsequent 24 h period. Quantification of cell-secreted Aβ_40_ was determined by ELISA, as detailed in [10].

### 4.7. Data Analysis

Data were analysed using Graphpad Prism (v6.01, La Jolla, CA, USA) and Microsoft Excel 2019. Values are expressed as mean ± SEM. Calcein-AM and ELISA data were statistically analysed using one-way ANOVA, followed by Tukey’s and Dunnett’s tests, respectively. Western blot quantification was statistically analysed using Student’s *t*-test. A *p*-value of 0.05 was considered significant (* *p* ≤ 0.05, ** *p* ≤ 0.01).

## Figures and Tables

**Table 1:** Intracellular calcein accumulation rates were calculated using the fluorescence data obtained over the final five minutes of the calcein-AM assay as depicted in Figure 1c. Data (mean ± SEM) are representative of three independent experiments, each performed with eight biological replicates. ** *p* ≤ 0.01 compared with control. † *p* ≤ 0.01 compared with Nedd4 siRNA-treated cells.

**Treatment**	Nedd4 siRNA	Nedd4 siRNA + Verapamil	Control siRNA	Control siRNA + Verapamil
**Rate constant (r.f.u./min)**	481 ± 55 **	627 ± 78 †	596 ± 66	740 ± 43 **

## Abbreviations

Aβ	Amyloid-beta
ABC	ATP-binding cassette
AD	Alzheimer’s disease
APP	Amyloid precursor protein
BBB	Blood-brain barrier
Calcein-AM	Calcein-acetoxymethyl ester
CHO	Chinese hamster ovary
DMSO	Dimethyl sulfoxide
ELISA	Enzyme-linked immunosorbent assay
HBSS	Hanks’ Balanced Salt Solution
HECT	Homologous to the E6-AP Carboxyl Terminus
NEDD4-1	Neural precursor cell-expressed developmentally downregulated protein 4-1
P-gp	P-glycoprotein
PBS	Phosphate buffered saline
siRNA	Small interfering ribonucleic acid
